# Application of the Endoscopic Micro-Inspection Tool QEVO® in the Surgical Treatment of Anterior Circulation Aneurysms—A Technical Note and Case Series

**DOI:** 10.3389/fsurg.2020.602080

**Published:** 2020-11-24

**Authors:** Karl-Michael Schebesch, Christian Doenitz, Amer Haj, Julius Höhne, Nils Ole Schmidt

**Affiliations:** Department of Neurosurgery, Medical Center of the University of Regensburg, Regensburg, Germany

**Keywords:** QEVO®, micro-inspection tool, neuro-endoscopy, cerebral aneurysm, aneurysm clipping, vascular neurosurgery, KINEVO

## Abstract

**Introduction:** The application of neuro-endoscopes in cerebral aneurysm surgery may help to avoid unintended aneurysm remnants and the accidental clipping of perforating arteries and aid the detection of blood collecting in the subdural spaces. Here, we present our experience with the novel endoscopic micro-inspection tool QEVO® (Carl Zeiss Meditec, Germany) in aneurysm surgery.

**Materials and Equipment:** In all patients the surgical microscope KINEVO® (Carl Zeiss Meditec, Germany) and the Microinspection tool QEVO® were applied.

**Methods:** The case series comprises 22 unruptured cerebral aneurysms of the anterior circulation. All aneurysms were treated surgically. All patients routinely underwent computed tomography and digital subtraction angiography within 10 days after surgery.

**Results:** No aneurysm remnants, cerebral ischemic deficits, or subdural hematomas were detected.

**Discussion:** In this technical note, we discuss the benefits and limitations of the QEVO® tool and illustrate the major paradigms by means of intraoperative photographs.

## Introduction

Endoscopes can be used in the surgical treatment of anterior circulation aneurysms, mainly because of benefits such as minimizing the extent of the surgical approach and craniotomy (1, 2), facilitating direct access to the aneurysm without dissecting the Sylvian fissure (2, 3), improving visualization of the perforating arteries (4, 5), depicting hidden structures such as the area below the anterior perforated substance and the optic tract (5), decreasing the risk of accidental brain damage (3), and meticulously visualizing the cranial nerves (6).

In 2004, Profeta et al. explored a rigid 30°-angle endoscope over a 3-year period and found improved overall safety, especially in aneurysms concealed behind the parent artery or when the view toward the major vessels was obscured by the dome (7). The sophisticated cadaveric study presented by Albert L. Rhoton Jr. et al. in 2014 showed that endoscopes significantly improve and complete the microscopic view of the surgical field (5).

So far, the majority of literature reports have solely involved the application of conventional, fixed-angled, and rigid endoscopes, well-known from their use in transsphenoidal surgery. Without exception, every endoscope has to be draped and connected to a video tower, thus increasing the number of devices in the operating theater. Besides, the use of more than one optical modality increases the inconvenience to surgeons because they are forced to always switch between the oculars of the microscope and the screen of the video tower (7).

In contrast, the innovative micro-inspection tool QEVO® (Carl Zeiss Meditec, Germany) is a small, rigid, un-draped, and fixed 45°-angle endoscope that can be easily connected to the surgical microscope KINEVO® (Carl Zeiss Meditec, Germany) in a plug-and-play fashion (8). Neurosurgeons may actively choose between displaying the endoscopic picture on the screen of the KINEVO® surgical microscope or looking directly into the oculars of the microscope.

Here, we present our first experiences with the QEVO® endoscope in the surgical treatment of anterior circulation aneurysms. In our neurosurgical department we didn't routinely use endoscopic surgical techniques in vascular surgery until applying the QEVO® endoscope. We categorized scientific assessment into “anatomical overview” at the beginning of aneurysm dissection, “patency of clip and vessels” after clip application, and inspection of the “subdural space” at the end of surgery. To the best of our knowledge, this is the first report about this specific endoscopic technical adjunct in vascular neurosurgery.

## Materials and Equipment

**Figure 1:**
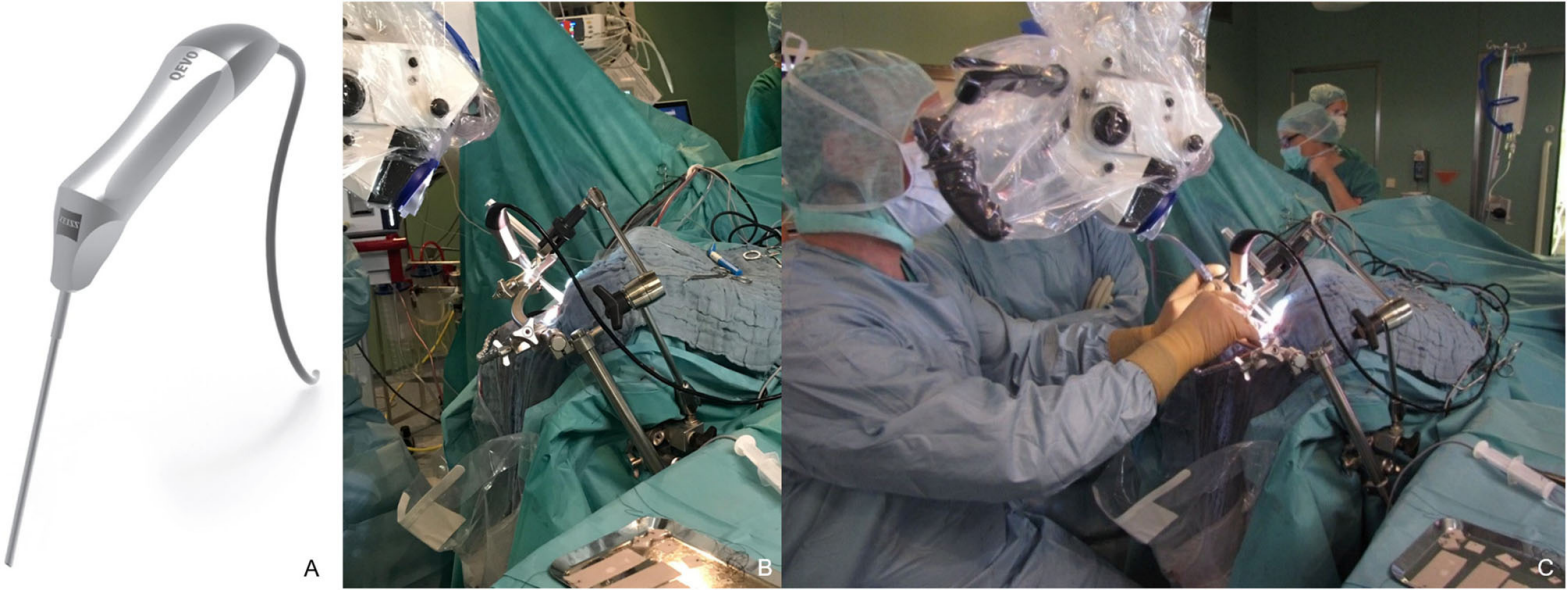
QEVO® micro-inspection tool **(A)** and setup in the operating theater, **(B)** mounted self-constructed endoscope-holder, **(C)** simultaneous visualization with the surgical microscope KINEVO® and QEVO® during microsurgical dissection.

For this technical report, we exclusively used the QEVO® micro-inspection tool—an undraped, external plug-and-play device that is solely featured by the KINEVO® surgical microscope. Both devices have been described in detail elsewhere (8). Briefly, the QEVO® endoscope is a rigid endoscope measuring 12.0 cm in length and 3.6 mm in thickness that allows a fixed 45°-angle view (Figure 1A). The endoscopic image is digitally transferred to the internal screen of the surgical microscope or can be alternatively displayed in the oculars of the KINEVO® surgical microscope.

## Methods

This study included 16 consecutive patients (11 women, 5 men, mean age 58 years) with 22 unruptured cerebral aneurysms of the anterior circulation that were surgically treated after interdisciplinary decision. Videos of all applied optical devices were stored for each procedure (QEVO® and KINEVO®). The sites of the aneurysms were distributed as follows: Middle cerebral artery (MCA, *n* = 17), anterior communicating artery (Acomm, *n* = 3), anterior cerebral artery (ACA, *n* = 1), and pericallosal artery (PA, *n* = 1). The most educative cases were selected as representative case illustrations for this report.

Firstly, the QEVO® endoscope was inserted during or after opening the Sylvia fissure or the interhemispheric approach to assess the endoscopic overview of the aneurysm and its surrounding vasculature, cranial nerves, and parenchyma. The second application of the QEVO® endoscope was before and after the clipping of the dissected aneurysm, and the third application consisted of the insertion into the subdural spaces to rule out any remote hematomas.

In three cases, a self-constructed endoscope-holder had been fixed to the head clamp (Figure 1B), allowing simultaneous microscopic and endoscopic visualization of the surgical field. The endoscope-holder was adjusted accordingly to aid further dissection (Figure 1C).

Besides evaluating general issues such as light and picture quality, ergonomics, heat development, and compatibility with the self-constructed endoscope-holder, we examined the usefulness and efficacy of the QEVO® endoscope in displaying subdural spaces and aneurysm environments but also in detecting hidden, tiny structures such as perforators, passing and branching vessels, and the tips of the clip(s).

According to our institutional standard, all patients underwent postoperative computed tomography (CT) within 24 h after surgery to rule out any surgery-related complications. Furthermore, each patient received early postoperative digital subtraction angiography (DSA) within 10 days after clipping to confirm aneurysm occlusion.

The study was approved on by the local Ethical Review Board (7-23-2020; reference: 20-1951-104).

## Results

In all surgical procedures, the QEVO® endoscope was connected to the KINEVO® surgical microscope with a cable and thus readily available for the remaining surgical time. The external light source of the QEVO® endoscope was switched on by pressing a button on the microscope's handgrip, so that the endoscopic picture became immediately visible on the general screen for everyone in the operating theater. The image quality was excellent at all times, and the light intensity was consistently adequate. Heat development in the corpus of the QEVO® endoscope was negligible. In summary, the handling of the micro-inspection tool was comfortable for the surgeon and did not interrupt the surgical flow as it would be the case when using a conventional endoscope.

The application of the QEVO® endoscope resulted in repositioning of the aneurysm clip in three cases (19%). In two patients, the initial position of the clip did not occlude the aneurysm base properly, and in one patient the clip significantly affected a small perforator. In this case series no aneurysm remnant was detected on postoperative DSA, and the early postoperative CT scan did not reveal any perfusion deficit or surgical complication. Consecutively, no patient developed a new neurological deficit.

### “Anatomical Overview”

Endoscopes are used to significantly enhance the conventional microscopic view of aneurysms and their surrounding vessels and tissues. The encasement of an aneurysm in the subarachnoid layers and its relationship to the adjacent vessels becomes clearly visible, and the view angle from behind or below the visual axis of the microscope helps to detect concealed anatomical details.

### Case Illustrations I-III for “Anatomical Overview”

**Figure 2:**
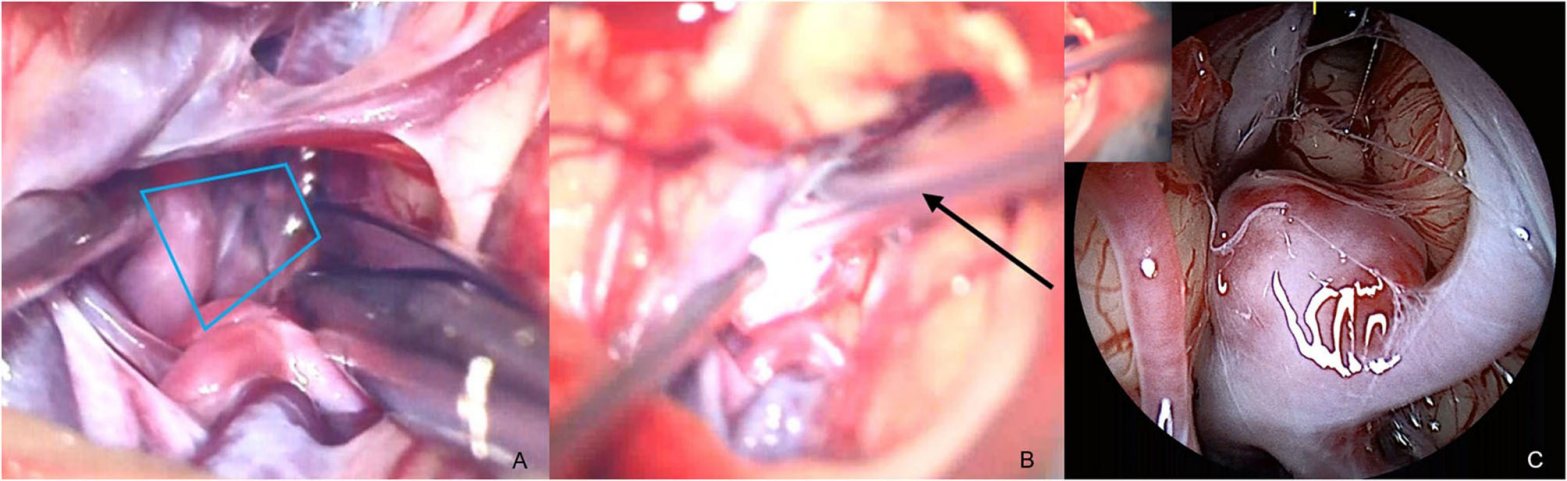
Anatomical overview, case I **(A)** opening of Sylvian Fissure/microscope, **(B)** insertion of the QEVO® endoscope (black arrow)/microscope, **(C)** aneurysm in arachnoid layers and surrounding structures/endoscope. *The blue trapezium in* ***(A)****represents the field of view of the QEVO*® *endoscope* ***(C)****as shown with the QEVO*® *endoscope in position in* ***(B)***.

Case I, Figures 2A–C. Left MCA aneurysm. The aneurysm was microscopically approached through the Sylvian fissure (Figure 2A), and the QEVO® endoscope was inserted from anterior and below the aneurysm (Figure 2B). The corresponding endoscopic view shows the subarachnoid encasement of the dome, the relationship to the parent vessel, and the position of the aneurysm between the temporal and the frontal lobe (Figure 2C).

**Figure 3:**
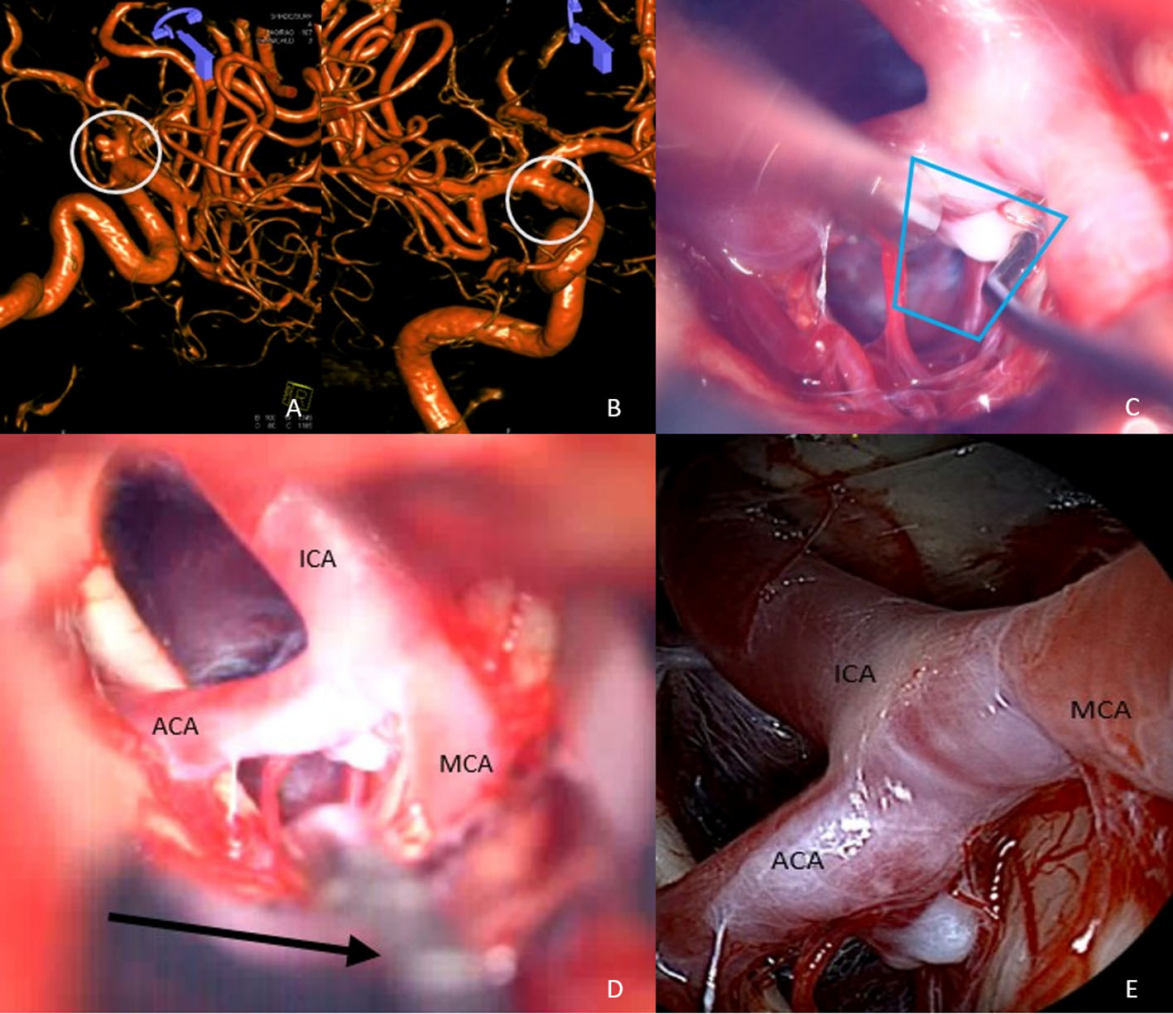
Anatomical overview, case II **(A,B)** 3D angiography of a right ACA aneurysm, **(C)** dissection of aneurysm/microscope, **(D)** insertion of QEVO® endoscope (black arrow)/microscope, **(E)** ICA and ACA from below with adjacent perforators/endoscope. *The blue trapezium in* ***(C)****represents the field of view of the QEVO*® *endoscope* ***(E)****as shown with the QEVO*® *endoscope in position in* ***(D)***.

Case II, Figures 3A–E. Right ACA aneurysm. The small aneurysm is projected on the back of the proximal A1 segment as shown by means of 3-dimensional angiography (Figures 3A,B). The anterior tip of the aneurysm can be made visible microscopically, and a tiny perforator is carefully translocated (Figure 3C). The QEVO® endoscope was inserted anterior of the ACA to inspect the aneurysm base which was not visible by microscope (Figure 3D).

**Figure 4:**
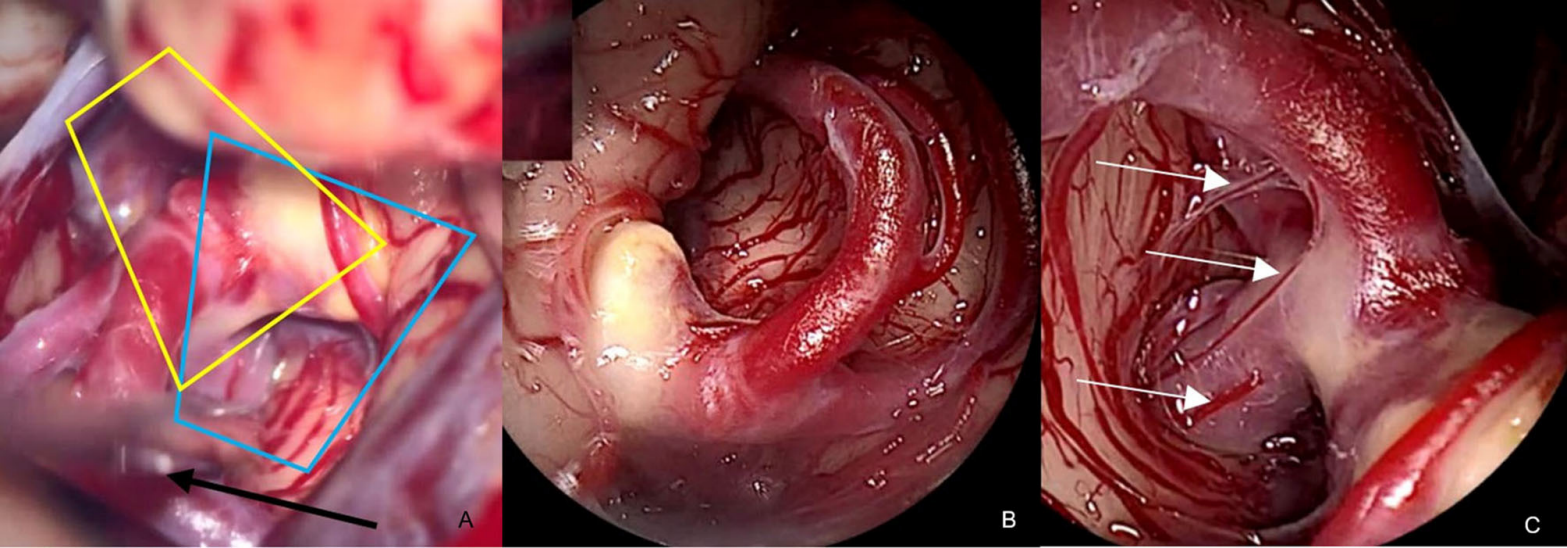
Anatomical overview, case III **(A)** partially dissected aneurysm base and QEVO® endoscope (black arrow)/microscope, **(B)** inspection of the aneurysm from above/endoscope, **(C)** inspection of the aneurysm from behind and below. Please note: perforators on the back of the MCA main branch (white arrows). *The blue trapezium in* ***(A)*** *represents the field of view* ***(B)****of the QEVO*® *endoscope in position as shown (black arrow) in* ***(C)****. The yellow trapezium in* ***(A)****represents the view from behind and below the aneurysm as shown in* ***(C)***.

Case III, Figures 4A–C. Left MCA aneurysm. Splitting of the Sylvian fissure and partial dissection of the aneurysmal basis (Figure 4A). The endoscopic view from anterior and below does not show any perforating arteries adjacent to the aneurysm (Figure 4B), but insertion of the QEVO® endoscope behind the aneurysm shows some hidden perforators on the back of the aneurysm (Figure 4C).

### “Patency of Clip and Vessels”

Directly before and immediately after clipping the dissected aneurysm, we inspected the relevant areas of interest with the QEVO® endoscope, in addition to micro-Doppler sonography and real-time intraoperative angiography with indocyanine green (ICG) or fluorescein sodium (FL) and the respective filters. The most important questions at this stage of surgery were whether the tips of the clip surmounted the aneurysm and occluded it completely, and, even more important, whether small branching perforators and/or en-passant vessels were separated and patent and not accidentally affected by the clip.

### Case Illustration IV-VI for “Patency of Clip and Vessels”

**Figure 5:**
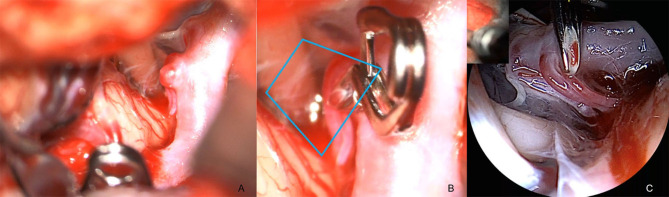
Patency of clip and vessels, case IV **(A)** view of the dissected side-wall aneurysm with two small adjacent perforators/microscope, **(B)** inspection of the aneurysm after clip application. The two perforators remain unchanged in size and color; however, the clip tips could have potentially affected the hidden parts of the vessels, **(C)** retrograde view of the QEVO® endoscope confirming complete occlusion of the aneurysm with still perfused perforators. *The blue trapezium in* ***(B)****represents the field of view of the QEVO*® *endoscope as shown in* ***(C)***.

Case IV, Figures 5A–C. Left MCA aneurysm. After the dissection of the small side-wall aneurysm (Figure 5A), a mini clip was applied that seems to be well-positioned (Figure 5B). However, under the microscope, only Doppler ultrasound or fluorescence angiography may confirm the patency of the two branching arteries. The QEVO® endoscope positioned behind the clip shows that the perforating arteries are unaffected by the clip (Figure 5C).

**Figure 6:**
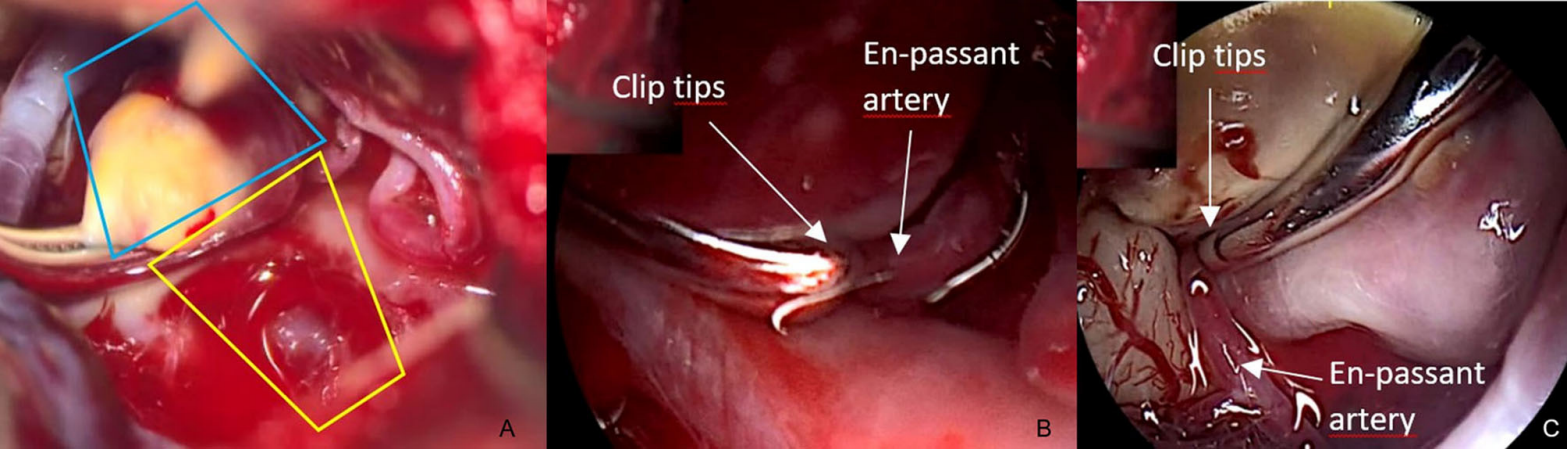
Patency of clip and vessels, case V **(A)** view on the clipped left-sided MCA aneurysm. The clip is partially concealed under the aneurysm sac/microscope, **(B)** close endoscopic inspection of the parietal brace confirms full patency of the en-passant artery, **(C)** close endoscopic inspection of the visceral brace confirms full patency of the en-passant artery. *The blue trapezium in* ***(A)****represents the field of view of the QEVO*® *endoscope as shown in* ***(B)****. The yellow trapezium in* ***(A)****is the corresponding field of view of the QEVO*® *endoscope as shown in* ***(C)***.

Case V, Figures 6A–C. Left MCA aneurysm. After application of the clip, the aneurysm sac deforms and partially conceals the clip (Figure 6A). With the QEVO® inserted posteriorly (Figure 6B) and anteriorly (Figure 6C), both clip braces are visualized, and the en-passant artery could be inspected closely.

**Figure 7:**
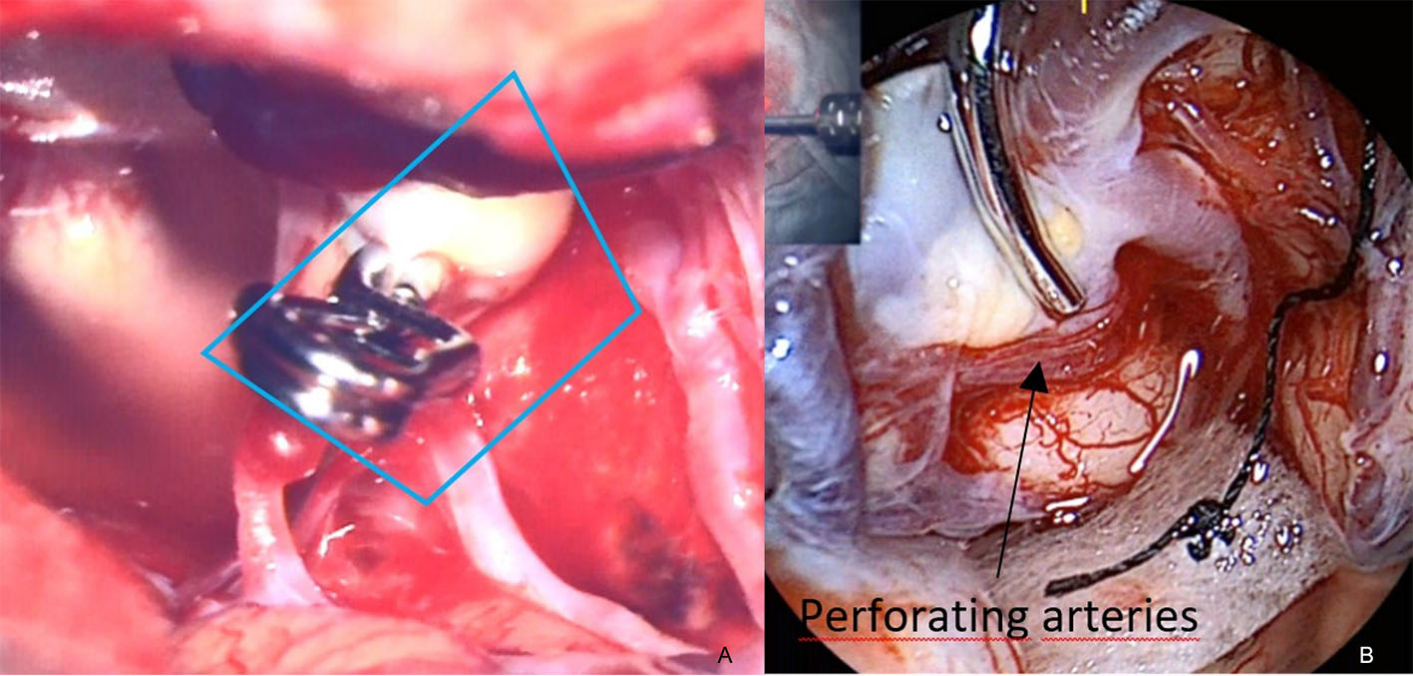
Patency of clip and vessels, case VI **(A)** microscopic view on the clipped aneurysm of the right-sided PA. Structures below the clip cannot be accurately evaluated, **(B)** endoscopic evaluation of the clip accuracy at the aneurysm base. The clip completely embraces the aneurysm, while the tiny perforators remain unaffected. *The blue trapezium in* ***(A)****represents the field of view of the QEVO*® *endoscope in* ***(B)***.

Case VI, Figures 7A,B. Right-sided aneurysm of the PA. After application of the clip, structures below the clip cannot be evaluated properly. With the QEVO® endoscope inserted behind (Figure 7A) and below (Figure 7B) the clip, the aneurysm base and a couple of tiny perforating arteries are visualized.

### Case Illustration VII for “Subdural Space”

**Figure 8:**
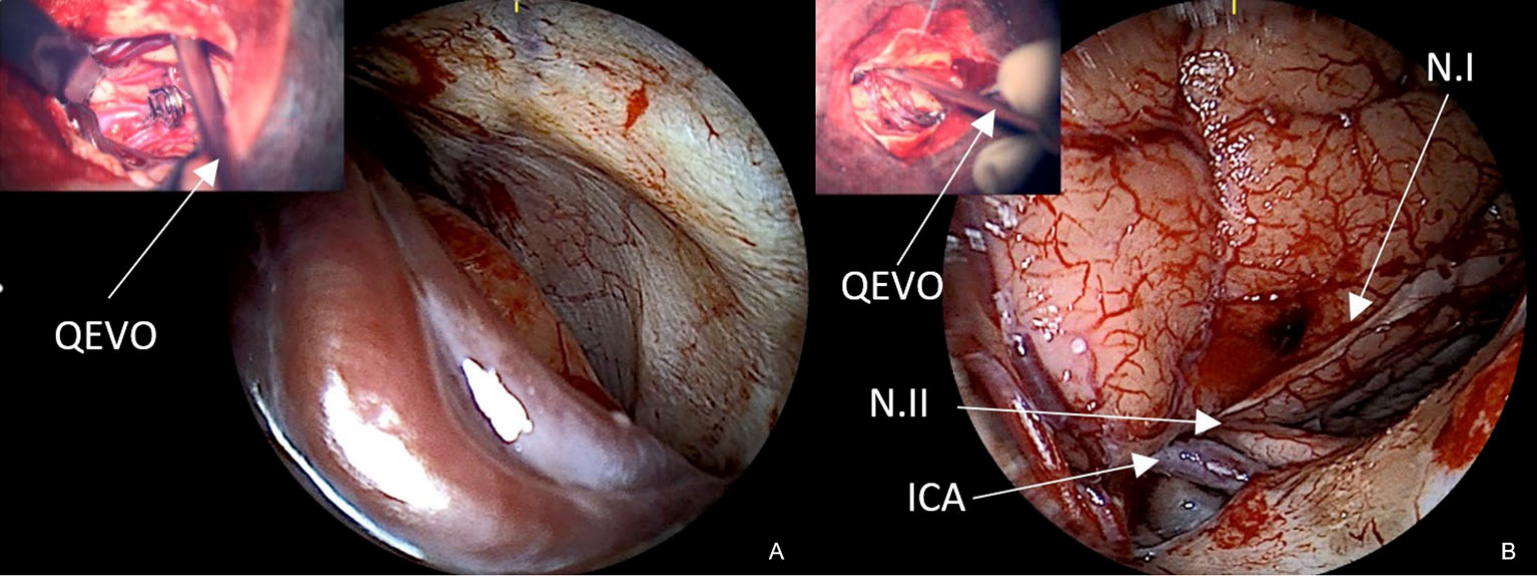
Subdural spaces **(A)** endoscopic evaluation of the left middle fossa with distal Sylvian veins to exclude remote hemorrhage, **(B)** endoscopic evaluation of the right parasellar region with ipsilateral ICA, N.I, N.II, and proximal Sylvian veins. The left upper pictures show the QEVO® endoscope *in situ* as seen under the microscope to illustrate its positioning.

At the end of microsurgery, we routinely check the accessible subdural spaces to exclude any blood clots or remote hematomas. With the surgical microscope and in the case of small pterional craniotomy, visualization of the subdural space is often not possible; however, the 45°-angle endoscope is an excellent and readily available tool for this purpose. Figure 8A shows the wide-open convexity of the basal temporal fossa and Figure 8B the subdural content of the right parasellar area. Both pictures were recorded at the end of surgery, confirming the absence of any remote hemorrhage or blood collection.

## Discussion

Modern neurovascular surgery aims to develop and perform safe and efficient surgical procedures in order to provide the best patient care. The aim of cerebral aneurysm surgery is to completely occlude the aneurysm while preserving all branching, passing, or adherent vessels avoiding severe neurological complications.

In recent years, the neurosurgical armamentarium has seen many significant technical advancements, for instance, visual and functional technical adjuncts such as the implementation of 3D-surgical microscopes, 3D-ultrasound, neuronavigation, fluorescence-guided techniques, and intraoperative neuro-monitoring. Nevertheless, a relatively high number of unintended aneurysm remnants are still detected after clipping (9, 10) and, even more important, aneurysm remnants with ischemic cerebral areas due to the accidental occlusion of small but considerable arterial perforators (11, 12). The application of a surgical endoscope significantly decreases the rate of postoperative ischemia (4, 5), and similar to our series, no patient was postoperatively diagnosed with aneurysm remnants or perfusion deficits.

Traditionally, micro-Doppler sonography was used to evaluate flow patterns inside aneurysms and their surrounding vasculature (13). In 2005, the results of the prospective indocyanine green trial in aneurysm surgery by Raabe et al. extended intraoperative blood flow assessment, and this method has now partially replaced micro-Doppler sonography (14). However, the application of micro-Doppler sonography and ICG angiography is limited to the circumscribed viewing field of the surgical microscope, and ICG angiography is only feasible in illuminated areas. Thus, obscure areas and hidden structures may only be detected by risky movements (15).

Therefore, an additional endoscope that is able to depict tissue and vessels behind, below, and above the aneurysm would significantly improve optical modalities, allowing a close and comprehensive view of the entire surgical field (5). In this study, we aimed to support the sparse data available on the beneficial use of endoscopes in aneurysm surgery for the morphological assessment of the vasculature and aneurysm as well as the subarachnoid and subdural spaces.

Neuro-endoscopy has a long tradition (16, 17), and many technical and haptic innovations and improvements have helped to establish endoscopes as tools that enable neurosurgeons to critically evaluate obscure anatomical areas, which is particularly important in complex aneurysm surgery (18, 19).

Some groundbreaking cadaver studies as well as clinical studies have shown the huge potential of endoscopically-assisted neurosurgery. In particular, the following advantages have been outlined: (1) minimizing the extent of the surgical approach and craniotomy (1), (2) reducing the risk of accidental brain damage (2), (3) avoiding Sylvian fissure dissection in Acomm-aneurysm surgery (3), and (4) meticulously visualizing the cranial nerves in pre-pontine aneurysm surgery via the cerebello-pontine angle (6). However, using a conventional endoscope during microsurgery disrupts the surgical flow due to the separate handling of the endoscope and its separate visualization on an external monitor.

A real innovation in this respect is the micro-inspection tool QEVO® that has been designed as a simple plug-and-play endoscope in combination with the next-generation KINEVO® surgical microscope. Thus, no video tower is needed because the endoscope image is displayed on the monitor of the microscope. The haptic features and technical aspects have already been evaluated elsewhere (8). Besides the bright white light illumination and the 4K image resolution, this tool has various advantages over conventional endoscopes, for instance a fixed 45° angle and displaying the image in the oculars of the specific microscope so that surgeons do not have to look up during the procedure. The probe that strongly resembles a surgical suction device does not have to be additionally draped because it is fully sterilizable.

Another important issue that we encountered during the first applications of the QEVO® endoscope is the possibility to provide a comprehensive anatomical overview at the beginning of aneurysm dissection. The initial assessment of otherwise concealed vessels, tissue, and arachnoid adherences behind or below the aneurysm dome may further facilitate circumferential preparation and help to avoid hazardous surgical movements (4). Consequently, in our series, the application of the QEVO® resulted in repositioning of the initial aneurysm clip in 19% (*n* = 3). Secondarily, we assume that collecting morphologic details of areas that are difficult to access during the initial phase of dissection may significantly speed up surgery times.

Finally, use of the micro-inspection tool QEVO® to visualize remote subdural spaces at the end of surgery may also increase the safety profile. As demonstrated in our case series, an extensive view alongside the temporal base helps to rule out the presence of blood clots or remote hematomas.

However, despite the discussed technical and assumedly beneficial features, there is still potential to further improve and accelerate endoscopic assistance. The following three developments should be addressed in the future: (1) the development of an endoscopic angiographic tool to simultaneously assess ICG and/or fluorescence-enhanced blood flow, (2) the development of a conventional endoscope-holder to facilitate and speed up surgery time, (3) development of a rotatable endoscope shaft, and (4) increase in the modifiable angle up to 180° to improve efficacy.

Hashimoto et al. successfully evaluated a 30°-angle neuro-endoscope capable of capturing intraoperative video-angiography to show flow patterns in the dead angles of the microscope. This device allowed the best possible assessment of anatomy and perfusion in the periphery of aneurysms, which led to reapplication of the clip in two patients (15). Catapano et al. included 40 patients in a prospective study evaluating the feasibility and the potential benefit of an ICG-endoscope. The authors concluded that this innovative technique enhances the surgical armamentarium significantly (20).

Regarding the development of a rigid variable-view endoscope, Ebner et al. published first experiences in a cadaver study using an adjustable field of view of up to 270°. The authors concluded that a variable endoscopic angle significantly improves surgical conditions (21). Cavallo et al. reported their experiences in applying a multidirectional video-endoscope with a modifiable angle (0 and 70 degrees) in a variety of neuro-endoscopic procedures. Optimized visibility and maneuverability were identified as the major benefits of this versatile tool (22).

A rotatable endoscope shaft would further enhance the surgical impact of the QEVO®, especially in small surgical corridors where instruments tend to be clustered and the camera head may be in the line of sight. Currently, the QEVO® shaft cannot be adjusted accordingly, and in some situations, it may require complete repositioning of the endoscope to the opposite side of the surgical field.

Finally, we designed and constructed an endoscope-holder that was connected to the head clamp to which the QEVO® probe was fixed. Although this self-constructed holder was practical, the interaction with the KINEVO® surgical microscope was partially cumbersome. Accordingly, we conclude that an endoscope-holder should be used when continuous endoscopic activity is warranted, for instance in skull base tumors. Endoscope-holders are dispensable in the case of short endoscopic evaluations of the surgical field such as in the present study.

In summary, despite some limitations, the micro-inspection tool QEVO® is a reliable and powerful method for enhancing the visual assessment of the surgical field in aneurysm surgery. This small endoscope enables the surgeon to magnify and illuminate structures in the depth of the surgical field, to look around corners and to confirm the patency of tiny perforating or branching vessels that bear a high risk of becoming accidentally affected. In our opinion, the QEVO® endoscope significantly enhances the surgical neuro-vascular armamentarium.

## Data Availability Statement

The raw data supporting the conclusions of this article will be made available by the authors, without undue reservation.

## Ethics Statement

The studies involving human participants were reviewed and approved by Ethical Committee of the University of Regensburg. Written informed consent for participation was not required for this study in accordance with the national legislation and the institutional requirements.

## Author Contributions

K-MS and NS designed the study and wrote the manuscript. CD, AH, and JH crafted and revised the manuscript. All authors contributed to the manuscript.

## Conflict of Interest

K-MS and JH have received honoraria, travels fees and scientific financial support from Carl Zeiss Meditec, Germany. The remaining authors declare that the research was conducted in the absence of any commercial or financial relationships that could be construed as a potential conflict of interest.
