# Supplementary figures and images for: Application of the Endoscopic Micro-Inspection Tool QEVO® in the Surgical Treatment of Anterior Circulation Aneurysms—A Technical Note and Case Series
Source: Front Surg. 2020 Nov 24;7:602080. doi: 10.3389/fsurg.2020.602080 (PMC7732632; doi:10.3389/fsurg.2020.602080)

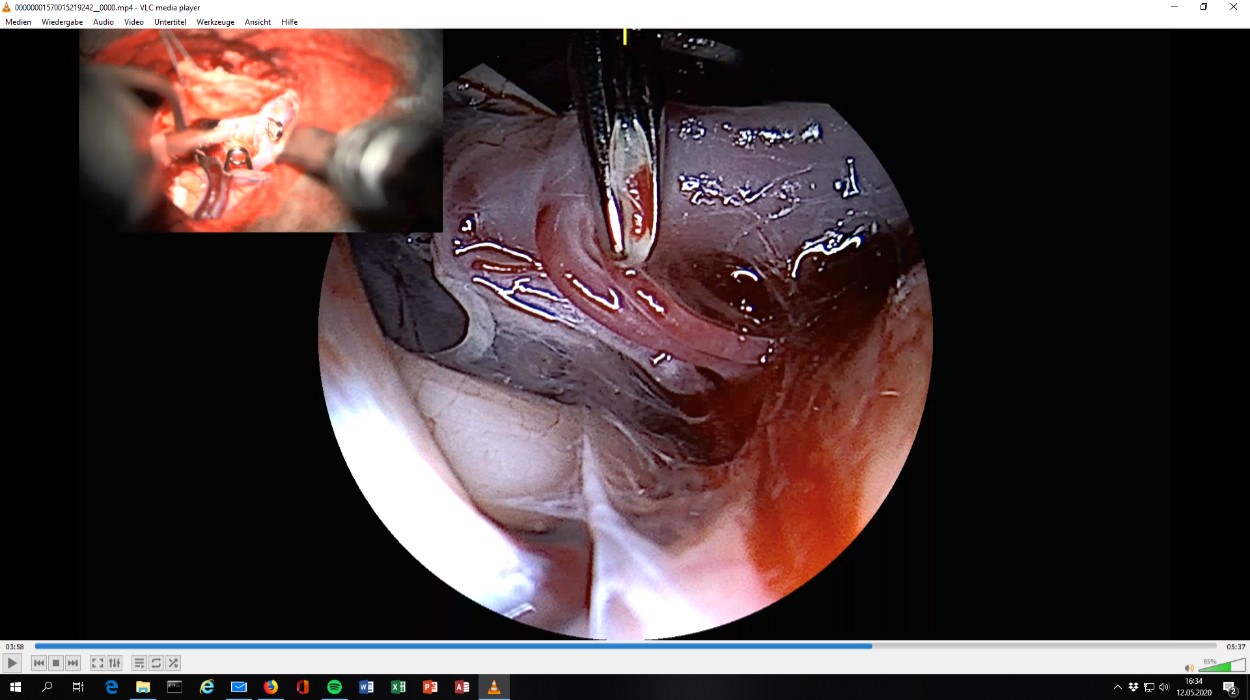

Supplement: Supplementary file 1 [file Image_1.JPEG]

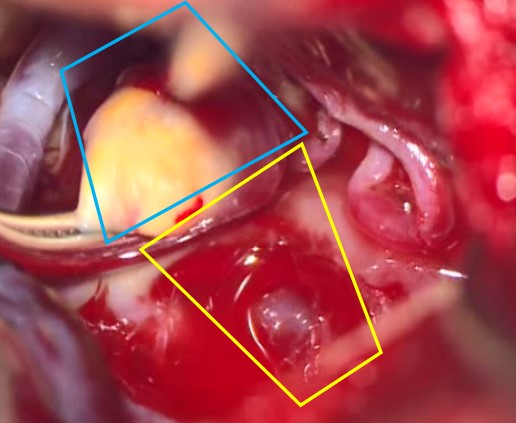

Supplement: Supplementary file 2 [file Image_2.JPEG]

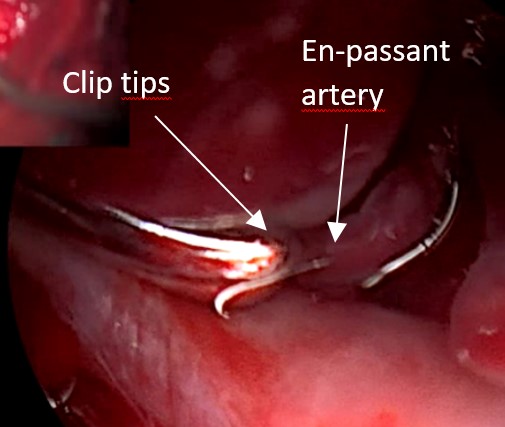

Supplement: Supplementary file 3 [file Image_3.JPEG]

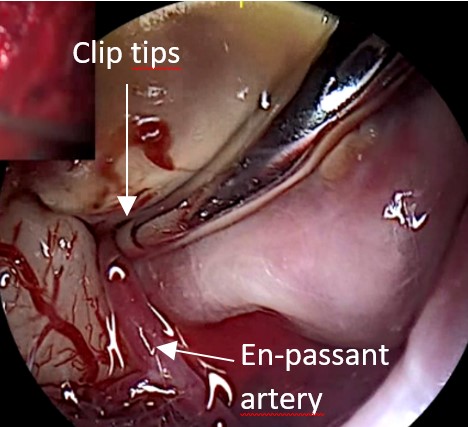

Supplement: Supplementary file 4 [file Image_4.JPEG]

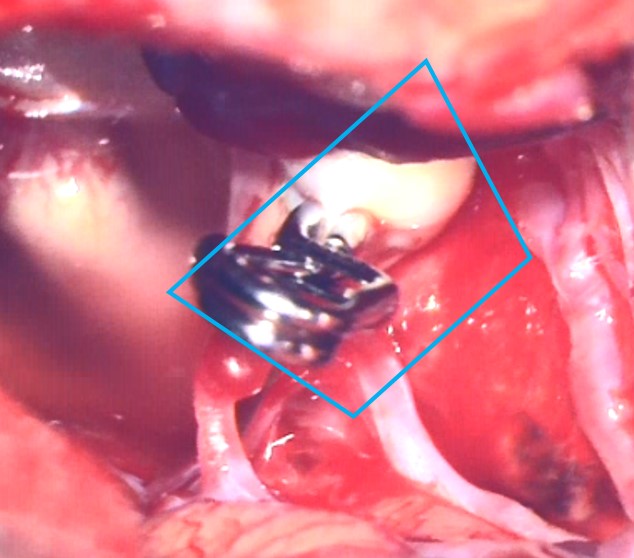

Supplement: Supplementary file 5 [file Image_5.JPEG]

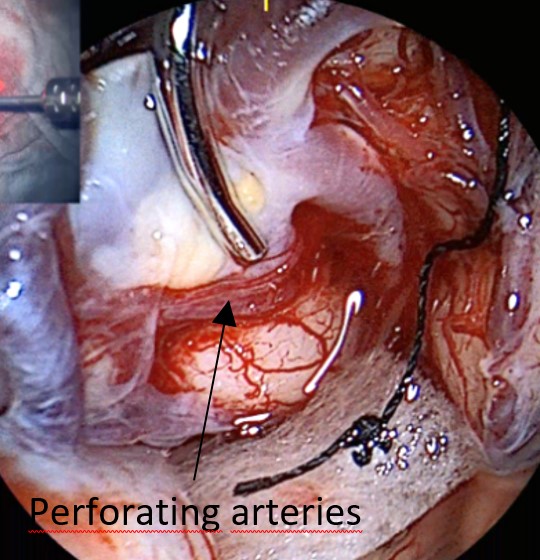

Supplement: Supplementary file 6 [file Image_6.JPEG]

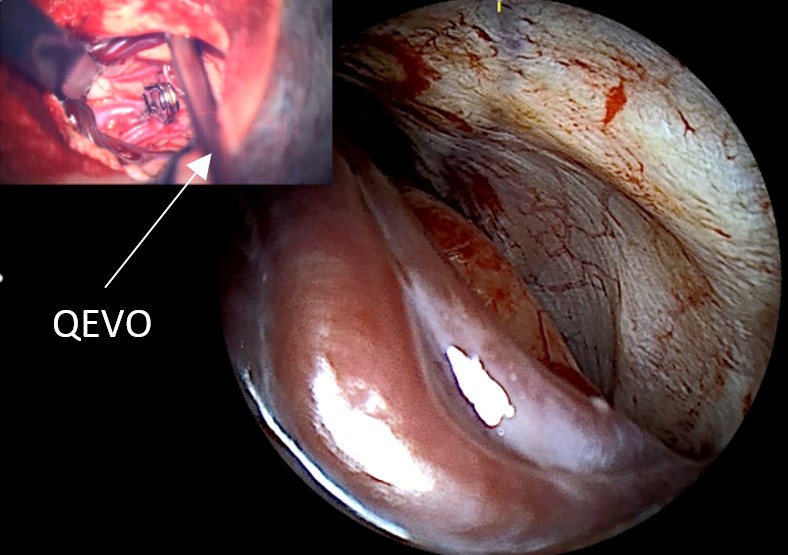

Supplement: Supplementary file 7 [file Image_7.JPEG]

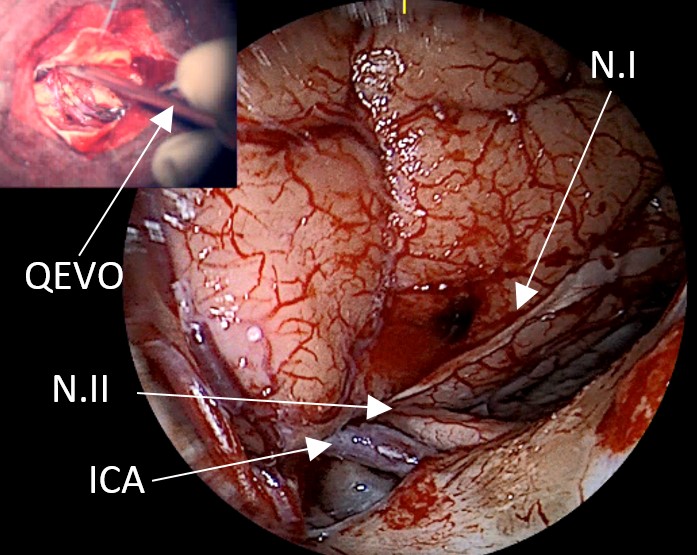

Supplement: Supplementary file 8 [file Image_8.JPEG]
